# Use of Minced Residual Skin Grafts to Improve Donor Site Healing in Split-Thickness Skin Grafting

**DOI:** 10.7759/cureus.23453

**Published:** 2022-03-24

**Authors:** Chandrashekhar Chalwade, Vineet Kumar, Aneesh Suresh

**Affiliations:** 1 Plastic Surgery, Nirmiti Clinic, Mumbai, IND; 2 Department of Plastic Surgery, Gordhandas Sunderdas (GS) Medical College and King Edward Memorial (KEM) Hospital, Mumbai, IND; 3 Plastic and Reconstructive Surgery, Tata Memorial Hospital and Homi Bhabha National Institute, Mumbai, IND; 4 Plastic and Reconstructive Surgery, Topiwala National Medical College and Bai Yamunabai Laxman (BYL) Nair Charitable Hospital, Mumbai, IND

**Keywords:** less scar, scar appearance, donor site, split-thickness skin graft (stsg), posas, epithelialization, minced residual skin grafting, donor site healing

## Abstract

Background

The morbidity of the donor site in split-thickness skin graft (STSG) may include abnormal pigmentation, delayed healing, and unfavorable scarring. Studies are usually focused on improving the healing of the recipient site, so donor site management becomes a secondary consideration. An optimal solution should be sought for donor site management to improve healing and minimize morbidity.

Methods

In this study, we used minced residual skin grafts over half of the donor site (cases) and compared the healing duration and scar quality with the other half (control). Healing duration was measured in days and the scar quality was assessed by the Patient and Observer Scar Assessment Scale (POSAS) at 90 days, 180 days, and 360 days.

Results

The healing time was reduced with the application of minced residual skin grafts on the donor site. The scar quality was significantly better in the case group as compared to the control group at 90 days, 180 days, and 360 days (p<0.05).

Conclusion

Mincing residual skin grafts and replacing them back to the donor site reduces the healing time and improves the quality of the scar.

## Introduction

Split-thickness skin grafting (STSG) is one of the most extensively performed procedures to achieve wound closure [1]. Most studies are focused on improving the appearance of scars on the recipient site and shortening the healing time, such that the management of the donor site usually takes a backseat. There is a lack of consensus with regards to the recommended method of donor site management [2-4].

Donor site morbidity of STSG is usually minimal, but that may not always be the case [5]. Morbidity may include pigmentary anomalies or dyschromia, prolonged pain, delayed healing, or unfavorable scarring [5]. So, an effort must be taken to find the optimal solution for donor site healing and minimize morbidity.

Small portions of the skin graft left at the end of the procedure or the graft obtained after trimming the edges are usually discarded. There have been only a few studies demonstrating the benefit of the minced residual skin graft on the donor site, resulting in earlier epithelialization and also improving the appearance [6-8]. With our study, we want to substantiate whether mincing residual skin grafting should be "routinely performed" to achieve optimal donor site healing.

This article was previously presented as an oral presentation at the 29th Annual Conference of the National Academy of Burns of India- NABICON 2021-22 on January 22, 2022.

## Materials and methods

Aims and objectives

The aim of this study is to assess the efficacy of minced residual skin grafts in improving donor site healing (duration of healing) and scar quality (Patient and Observer Scar Assessment Scale or POSAS score). The variables used to study the above-mentioned objectives were: (i) time taken to epithelialize the entire donor site (as assessed by observers) or duration of healing - expressed in number of days; and (ii) scar parameters at 90 days (three months), 180 days (six months), and 360 days (one year) as assessed by POSAS.

POSAS is a validated scar assessment scale that measures scar quality by evaluating visual, tactile, and sensory characteristics of the scar from two different perspectives: the patient perspective and the surgeons’ perspective [9]. The patient’s scale includes the following items: pain, itch, thickness, color, stiffness, and irregularity. The observer or clinician’s scale includes the following items: vascularity, pigmentation, texture, thickness, pliability, and surface area. Each item of the scale is rated on a 10-point score. The lowest score is 1 and the highest is 10. The total score on both scales can be calculated by adding up the scores of each of the six items. So, the total score ranges from 6 to 60. Lower scores indicate a situation closer to the normal skin whereas higher scores indicate a situation with a greater difference from the normal skin.

The authors assert that this study complied with the ethical standards of relevant national and institutional guidelines on human experimentation and the Helsinki declaration of 1975 (revised in 2008). Informed consent was obtained from all patients. This is an open-label, prospective, case-control study conducted for a period of one year (March 2020 to February 2021).

Inclusion and exclusion criteria

All patients who were posted for STSG and gave consent to participate in the study were included in the study. Exclusion criteria were as follows: (i) patients under the age of 18 years, ​​​​​​​(ii) patients with a known tendency for hypertrophic scarring/keloids, ​​​​​​​(iii) pregnancy, ​​​​​​​and (iv) co-morbid factors like uncontrolled diabetes, ischemic heart disease (IHD), renal failure, use of steroids. After evaluation of patients by the inclusion and exclusion criteria, suitable patients (n=30) were included in the study.

Surgical technique

Graft harvest was done under strict aseptic conditions using Watson's modification of Humby's knife with the graft thickness set at 0.15-0.30 mm. The size of the graft harvested was decided based on the wound requirements. The graft was secured over the wound, and the excess graft from the wound edges was trimmed. The residual graft was thoroughly washed with saline to prevent donor site contamination. 

These trimmed graft pieces, along with any unused grafts, were minced manually with blades and scissors into approximately 1 mm pieces. The donor area was divided into two approximate halves based on the number of 10 cm × 10 cm tulle dressings needed to cover the raw area. Hemostasis of the donor site was achieved, followed by the application of minced skin grafts.

In the case group, the minced skin graft was applied evenly over one-half of the donor site and covered with an occlusive non-adherent dressing in the form of a tulle and polyurethane foam, followed by an absorbent gamjee roll.

In the control group, the remaining half of the donor site was dressed with an occlusive non-adherent dressing alone in the form of a tulle and polyurethane foam, followed by an absorbent gamjee roll.

Postoperative management

After every dressing change session, photographic documentation was made. In the early postoperative period, the donor site dressing was assessed for soakage. If the dressing appeared soaked, the gamjee roll was gently replaced without disturbing the tulle and polyurethane foam dressing. Also, the donor site dressing was regularly assessed to see if there were signs of loosening, which was indicative of underlying epithelialization.

In our practice, we noticed that the donor sites were usually epithelialized by postoperative day (POD)-14 and that the dressings would slide off the donor sites on their own. With our intervention, we wanted to assess whether the application of minced skin grafts led to an earlier epithelialization. If the donor site dressing did not come off on its own, the first dressing change was done on POD-12 and subsequently every alternate day till complete epithelialization was noted in each group. The duration of healing was defined as the number of days required to achieve complete epithelialization.

The patients were followed up at 90 days (three months), 180 days (six months), and 360 days (one year) to assess for scar quality using the POSAS score. Patients were advised twice a day application of coconut oil or an emollient followed by a massage for 10 minutes (once in the morning and once in the evening), once the donor site had epithelialized.

Data analysis

Duration of healing, POSAS score, and other demographic parameters were recorded and analyzed using the OpenEpi calculator available online [10]. Duration of healing and POSAS score were represented as mean and standard deviation. A p-value of <0.05 was considered statistically significant.

## Results

Demographic characteristics

Our study included 21 males and 9 females. The average age of our study was 36.6 years. The average age of males was 43.2 years (range 18-67), whereas the average age of females was 30.8 years (19-50).

Duration of healing

A statistically significant difference (p-value = 0.0001) in the mean duration of healing time was noted for both groups. Healing time for the intervention/study group was shorter (14.93 ± 1.79 days with a range of 12-20 days) compared to the control group (16.13 ± 2.09 days with a range of 14-22 days).

**Figure 1:**
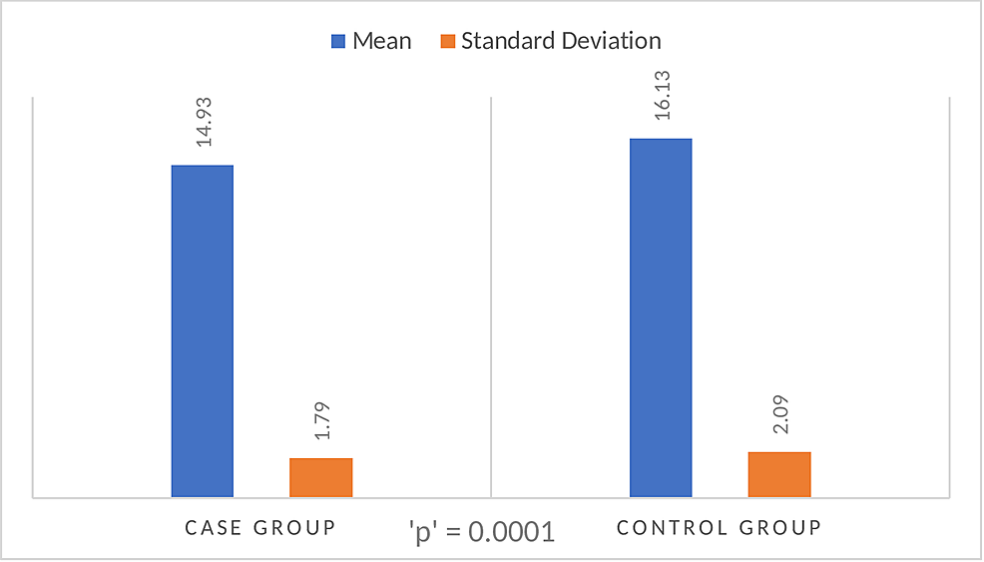
The mean duration of healing in the case group and the control group (in days).

Scar quality

Scar quality evaluation was assessed using POSAS at 90 days (three months), 180 days (six months), and 360 days (one year) from the date of surgery.

At 90 days, the mean in the case group when compared against the control group revealed lower values in both the observer (24.56 ± 6.17; R = 15.45 vs 28.53 ± 6.68; R = 13.46) and the patient scales (30.63 ± 8.93, R = 14.51 vs 35.56 ± 8.44, R = 16.53) with a 'p'-value of 0.02 and 0.03, respectively. Similarly, at 180 days, comparison of the mean in the case group against the control group revealed lower values in both the observer (20.1 ± 4.58; R = 11.32 vs 22.9 ± 5.28; R = 11.30) and the patient scales (22.83 ± 5.22, R = 13.34 vs 26 ± 5.31, R = 14.40) with a 'p'-value of 0.03 and 0.02, respectively. The same trend was noticed at 360 days, with a lower mean seen in the case group as compared to the control group in both the observer (6.2 ± 1.43, R = 3.12 vs 7.8 ± 1.82, R = 4.14) and the patient scales (8.9 ± 2.13, R = 5.16 vs 12 ± 2.32, R = 8.20) with a 'p'-value of 0.02 and 0.03, respectively.

**Figure 2:**
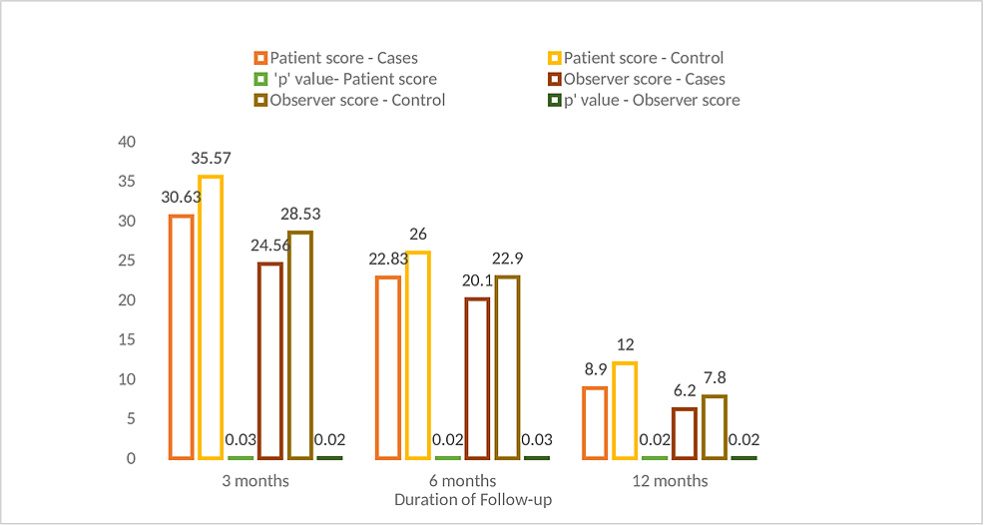
POSAS score at three months, six months, and one year. POSAS: Patient and Observer Scar Assessment Scale.

The POSAS score in the case group was significantly lower than that of the control group at three months, six months, and one year on the patient as well as observer scales, suggesting that there was an improvement in scar quality in the case group as compared to the control group and that the finding was statistically significant (Figure 2).

## Discussion

Historically, skin graft donor sites have been managed with different kinds of specialized dressings [2,3]. Such wounds heal by epithelialization. Epithelialization consists of the activation, migration, and proliferation of keratinocytes across the wound from the margin of the wound and residual epidermal appendages/adnexal structures like sebaceous glands, sweat glands, and hair follicles [11,12]. The healing of the donor site is proportionate to the available dermis and epidermal appendages. Hence, donor site healing is better after harvesting thin grafts rather than medium or full-thickness grafts. This process of wound healing might seem uneventful, but still includes the morbidity of pain and itching. Sometimes it may even be associated with abnormal pigmentation, erythema, and unfavorable scarring [5]. Despite STSG being a commonly performed procedure, there is a lack of consensus with regards to the recommended method of donor site management.

The concept of transplanting small parts of the skin onto wounds was probably first described by Reverdin as "pinch grafting" [13]. In this technique, STSG is divided over a set of parallel lamellas to create smaller pieces. The advantages included a theoretically high expansion ratio and the ability to perform this procedure with minimal resources. Similarly, the Meek technique created stamp grafts of 3 mm in size, which achieved greater expansion of the available skin. The disadvantages of the meek technique include a conspicuous patchy appearance and the need for specialized equipment to perform the technique [14,15]. Minced skin grafting or "chip" skin grafting was first reported by Harashina and Iso for the treatment of leukoderma [16]. They reported better aesthetic outcomes as compared to punch grafts. Simizu et al. were the first to report the use of minced residual graft at the donor site and found a favorable outcome [6]. Since then, only a few studies have reported its benefits for donor site healing [6-8].

We used the technique of mincing the unused and residual graft pieces trimmed from the edges of the recipient site. The duration of healing was significantly less in areas where the minced residual graft was applied than in areas where no graft was applied. Mincing led to a faster rate of epithelialization, possibly due to the release of keratinocytes, melanocytes, and stem cells in an environment of growth factors [7,8,17]. This correlates inversely to the size of the skin graft [17]. In summary, larger skin grafts behave like a "skin transplant," whereas the minced skin behaves like an "organ culture." Larger skin grafts would lead to re-epithelialization due to migration of keratinocytes from the edges of the graft, whereas minced grafting or micrografting promoted centripetal movement of the keratinocytes, melanocytes, and stem cells from the colonies, leading to greater re-epithelialization and pigmentation [17].

The improved appearance and reduced itchiness/pruritis at the donor site may be due to the release of growth factors such as interleukin-6 (IL-6), granulocyte-colony-stimulating factor (G-CSF), monocyte chemoattractant protein-1 (MCP-1), vascular endothelial growth factor (VEGF), and platelet-derived growth factor (PDGF) [18]. This may have led to reduced inflammation at the donor site and faster healing.

**Figure 3:**
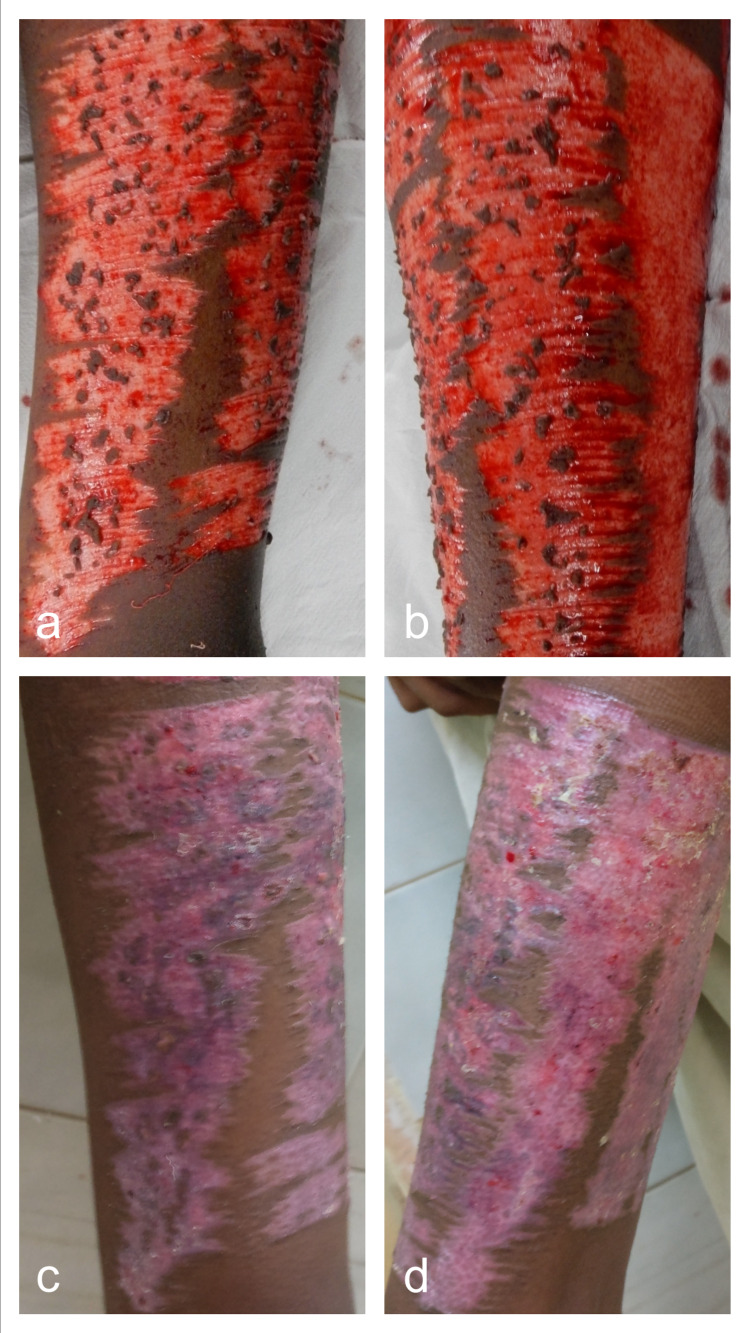
Intraoperative application of minced residual skin graft over half of the donor site (a) and the remaining half acting as the control (b). Anterior and medial thigh at an advanced stage of epithelialization and pigmentation (c) as compared to the lateral and posterior thigh at postoperative day-12 (d).

**Figure 4:**
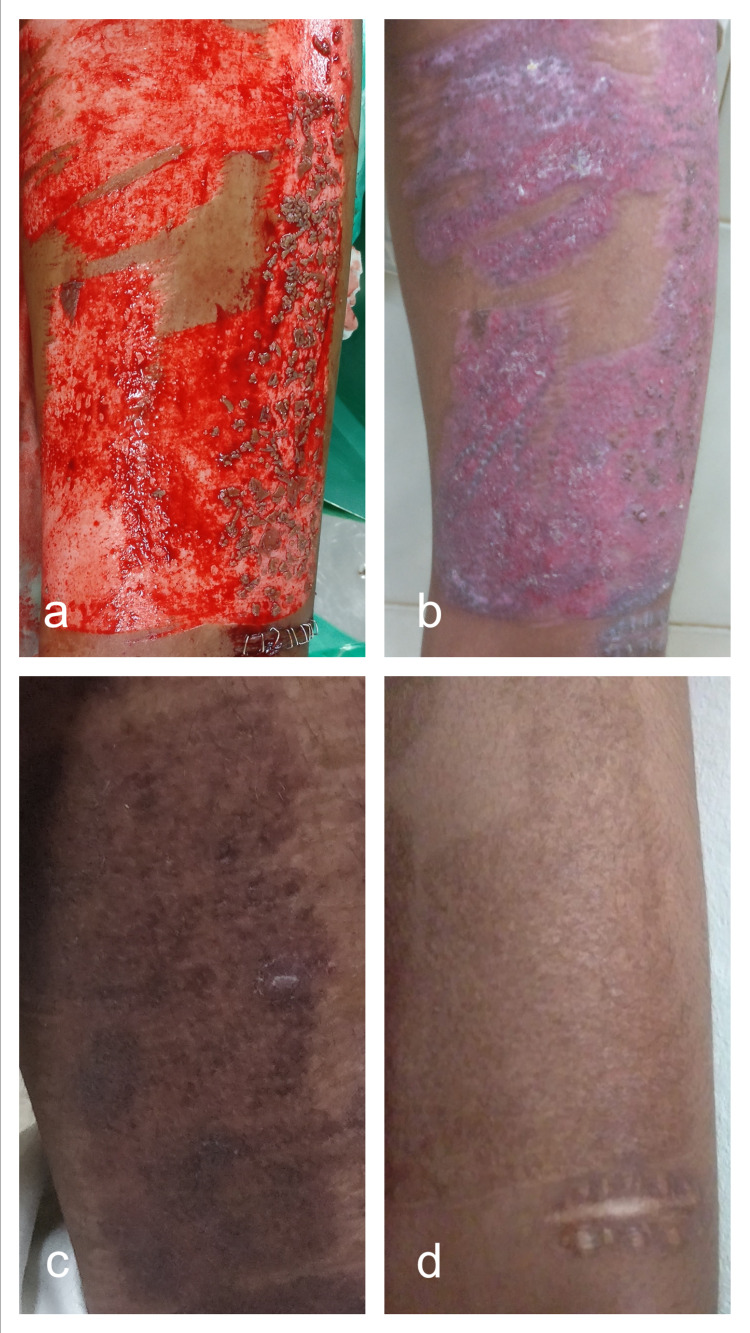
Donor site after application of minced residual skin graft intraoperatively (a) and appearance at postoperative day-12 (b). Medial thigh (control) showing hyperpigmentation (c) as compared to anterior thigh (case) showing good pigmentation and texture match as compared to the native skin (d).

**Figure 5:**
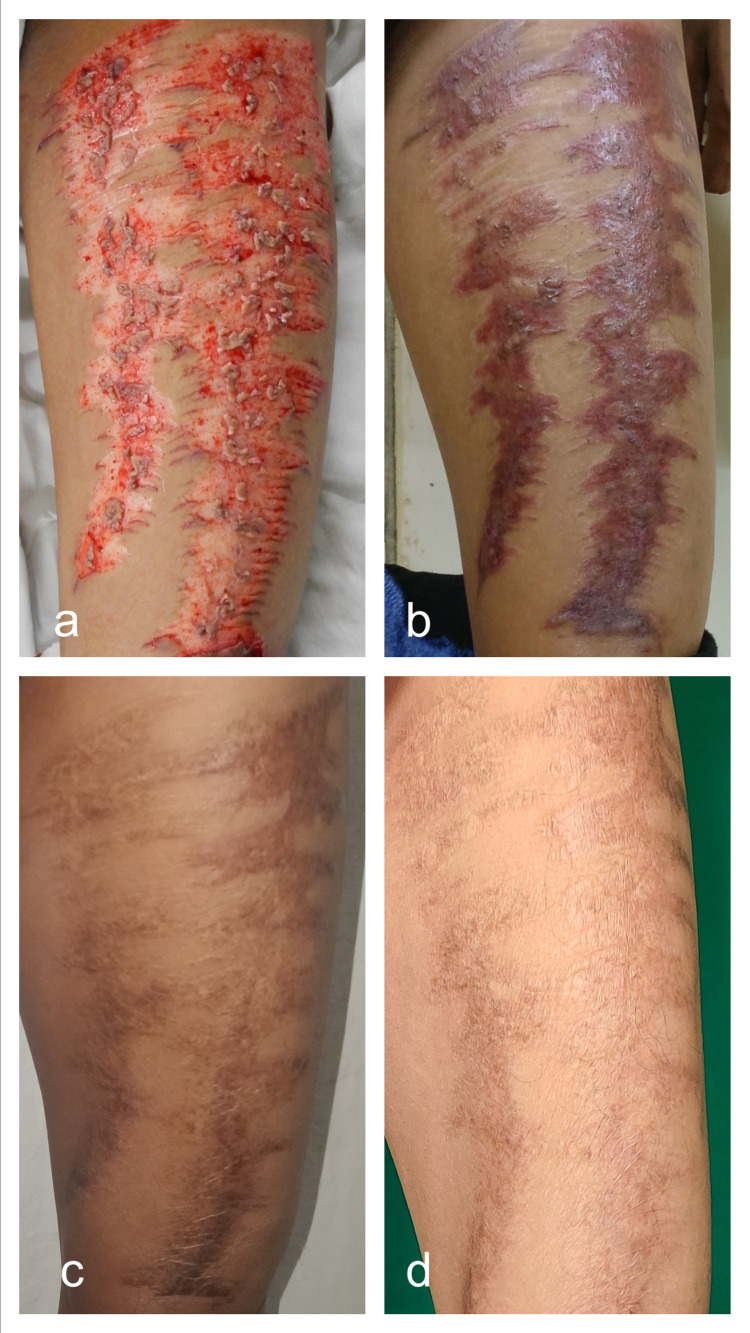
Donor site after application of minced residual skin graft intraoperatively (a) and appearance at three months (b), and at six months (c). Appearance at one year showing good color and texture match in comparison to the native skin (d).

**Figure 6:**
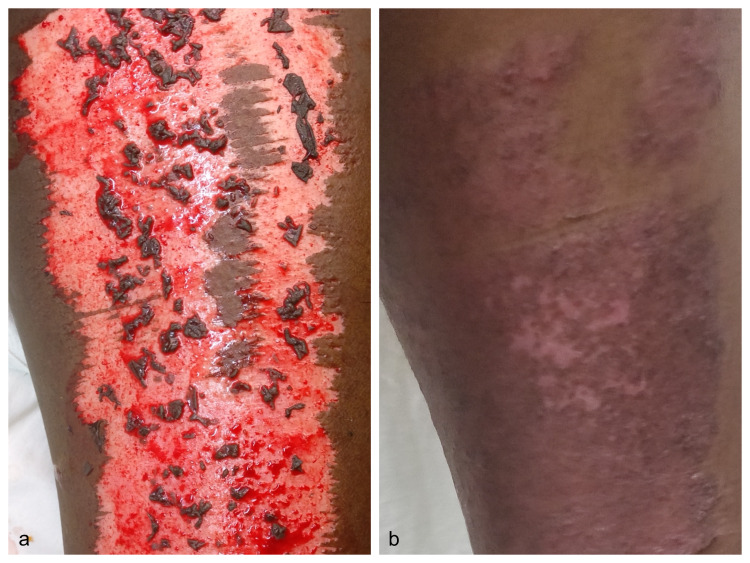
Donor site after application of minced residual skin graft intraoperatively (a) and appearance at one year showing healing with good pigmentation and skin texture (b).

Mincing the excess graft and replacing it on the donor sites reduced the time to heal and improved the scar quality (Figures 3-6). This finding was similar to the studies conducted by Simizu et al., Miyanaga et al., and Radharaman et al. [6-8].

**Figure 7:**
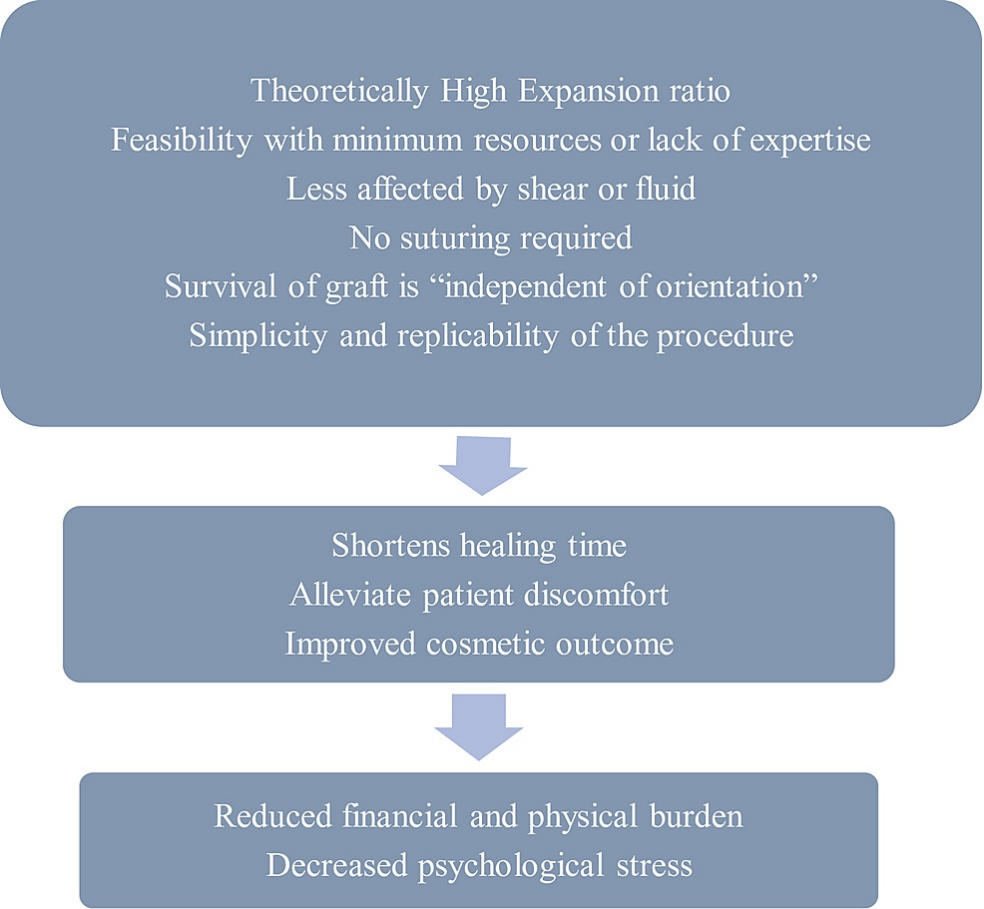
Benefits of residual minced skin grafting.

The excess grafts at the recipient site edges are either left or trimmed and discarded. So, this technique provides a win-win situation. The small graft pieces that otherwise would have been wasted or discarded are put to use in improving the donor site healing. The benefits or advantages of minced residual skin grafting have been shown in Figure 7.

The possible disadvantages of minced residual skin grafting might be that the procedure is more time-consuming as compared to performing no grafting. There is a risk of transmission of infection/contamination from the recipient site to the donor site. This can be prevented by thorough washing with saline before applying the residual grafts onto the donor site. Also, sometimes it may not be possible to cover the entire donor site with the residual minced grafting. We feel that despite these possible drawbacks, the advantages of the procedure far outweigh the disadvantages. As more studies contribute to the research, minced residual skin grafting can be advocated as a routine procedure following STSG.

Our study does have a few limitations. The sample size of our study was small. In our study, the available minced grafts were spread evenly over the case area without any particular ratio being observed. A further study with different ratios of spread might help in determining the optimum ratio, if any, to achieve faster healing. Also, there was no standardization of the control versus study site selection except for partitioning into two similar halves.

## Conclusions

The donor site of STSG treated with minced residual skin grafts showed a reduction in healing time and the healed scar had a significantly better appearance as compared to the donor site treated without a skin graft. Our study shows that, when applicable, this technique can be incorporated as a "standard procedure" following STSG to achieve optimal wound healing of the donor site.
